# Downregulation of TUSC3 promotes EMT and hepatocellular carcinoma progression through LIPC/AKT axis

**DOI:** 10.1186/s12967-022-03690-3

**Published:** 2022-10-23

**Authors:** Ruxia Deng, Xiansheng Lu, Chang Hong, Rui Cai, Ping Wang, Le Xiong, Xiaoyu Wang, Qiaoyu Chen, Jie Lin

**Affiliations:** 1 Department of Pathology, Nanfang Hospital, Southern Medical University, Guangzhou, 510515 Guangdong People’s Republic of China; 2 Department of Pathology,School of Basic Medical Sciences, Southern Medical University, Guangzhou, 510515 Guangdong People’s Republic of China; 3 Guangdong Province Key Laboratory of Molecular Tumor Pathology, Guangzhou, 510515 Guangdong People’s Republic of China; 4 Department of Pathology, Nanfang Hospital, Southern Medical University, Guangzhou, 510515 Guangdong People’s Republic of China; 5 Department of Pathology, School of Basic Medical Sciences, Southern Medical University, Guangzhou, 510515 Guangdong People’s Republic of China; 6 Guangdong Province Key Laboratory of Molecular Tumor Pathology, Guangzhou, 510515, Guangdong People’s Republic of China; 7 National Virtual & Reality Experimental Education Center for Medical Morphology, Southern Medical University, Guangzhou, 510515 Guangdong People’s Republic of China

**Keywords:** TUSC3, Epithelial–mesenchymal transition, LIPC, Hepatocellular carcinoma

## Abstract

**Background:**

Hepatocellular carcinoma (HCC) is one of the most common and malignant tumors in the digestive tract. Tumor Suppressor Candidate 3 (TUSC3) is one subunit of the endoplasmic reticulum Oligosaccharyl transferase (OST) complex, which plays an important role in N-glycosylation during the protein folding process. However, the role of TUSC3 in the initiation and progression of HCC has not been mentioned yet. In the present study, we aim to investigate the effects of TUSC3 on the initiation and progression of HCC.

**Methods:**

Immunohistochemical assay and qRT-PCR were used to detect the expression of TUSC3 and lipase C hepatic type (LIPC) in HCC tissue and cells. Loss-of-function and gain-of-function were applied to detect the function of TUSC3 and LIPC in vivo and in vitro. Immunofluorescence assay and co-immunoprecipitation were used to detect the relationship between TUSC3 and LPC. Western blot was applied to detect the expression of epithelial–mesenchymal transition (EMT) markers and the Akt signaling pathway.

**Results:**

TUSC3 was aberrantly decreased in hepatocellular carcinoma tissues compared to the matched adjacent normal tissues, which resulted in bigger size of tumor (P = 0.001, Table 2), worse differentiation (P = 0.006, Table 2) and an advanced BCLC stage. Down-regulation of TUSC3 led to the enhanced proliferation and migration of hepatocellular carcinoma cells in vivo and vitro, whereas the opposite effect could be observed in the TUSC3-overexpression group. The analysis of TUSC3 microarray showed that LIPC, a glycoprotein primarily synthesized and secreted by hepatocytes, was a downstream target of TUSC3, and it negatively modulated the development of HCC. The morphological changes in HCC cells indicated that TUSC3 regulated the epithelial-mesenchymal transition (EMT). Mechanistically, TUSC3 inhibited EMT progression through the LIPC/AKT axis.

**Conclusion:**

Down-regulation of TUSC3 promotes EMT progression by activating AKT signaling via targeting LIPC in HCC, which is probably the possible mechanism driving TUSC3-deficient hepatocellular carcinoma cells toward a malignant phenotype.

## Introduction

Hepatocellular carcinoma (HCC) is the most common primary hepatic cancer worldwide, with an ever-increasing prevalence [1]. The disease has the characteristics of latent onset, rapid progression, and high malignancy, indicating the dismal prognosis for HCC patients. According to the 2020 Global Cancer Statistics Report, liver cancer ranks the sixth in terms of cancer incidence and the third in mortality [2]. The treatment of HCC has been continuously improved in recent years. To some extent, targeted medicines and immunotherapy have improved the prognosis and survival time [3]. Apart from this, the recurrence and metastasis problems of hepatocellular carcinoma patients have not been effectively resolved [4]. Therefore, searching for applicable biomarkers is critical for the early diagnosis, timely treatment, and prognostic evaluation of hepatocellular carcinoma.

Tumor suppressor candidate 3 (TUSC3) plays an important role in N-glycosylation during the protein folding process by encoding one subunit of the endoplasmic reticulum (ER) Oligosaccharyl transferase (OST) complex, while the unfolded protein response (UPR) is carried on by its loss [5]. Therefore, TUSC3 gene was originally assumed to be a tumor suppressor candidate. For example, loss of TUSC3 expression in prostate cancer cells results in increased proliferation, migration, and invasion by affecting ER stress via Akt signaling [6]. In addition, loss of TUSC3 modifies the molecular response to ER stress and causes characteristics of the epithelial-to-mesenchymal transition (EMT) in ovarian cancer cells [7]. However, the development of cancer is a multi-step process, which involves several molecular events. For example, TUSC3 activates WNT/β-catenin and MAPK signaling to improve the proliferation and migration of colorectal cancer (CRC) cell lines [8]. Similarly, TUSC3 increases the proliferation of NSCLC cell lines via hedgehog (HH) signaling [9]. It is assumed that TUSC3 has a possible role beyond N-glycosylation. Moreover, role of TUSC3 in HCC has rarely been reported and deserves to be further investigated.

Lipase C hepatic type (LIPC) was the most related gene to TUSC3 by using TUSC3 microarray analysis. LIPC, a member of the lipase family, plays a key function in lipoprotein metabolism, and its aberrant expression has been linked to metabolic and cardiovascular disorders [10, 11]. Meanwhile, the aberrant expression of LIPC impacts the onset and progression of cancer, but its role in malignancies has not been concentrated on too much by researchers. In the context of non-small cell lung carcinoma (NSCLC), LIPC expression levels may have both a predictive value and an independent prognostic potential [12]. LIPC is highly expressed in a cohort of human hepatic metastasis and primary colorectal tumors [13, 14]. In addition, LIPC promotes the epithelial–mesenchymal transition (EMT) process in Borrmann type 4 gastric cancer [15] and pancreatic cancer [16]. It is indicated that LIPC is associated with tumor metastasis. EMT, a reversible cellular program that transforms epithelial cells into quasi-mesenchymal cell states, endows the tumor with several traits including tumor-initiation, which is essential to the malignancy [17]. Lipid metabolism can accelerate the procedure of EMT in the context of neoplasia, indicating the need for further studies to develop an effective tumor therapy [18, 19]. However, there are still no relevant reports about TUSC3, LIPC, and EMT in HCC.

In this study, we aimed to demonstrate the expression and roles of TUSC3 in HCC, and to explore the underlying mechanisms of TUSC3 in EMT and the progression of HCC.

## Materials and methods

### Tissue samples and ethical statement

This study was conducted based on the Declaration of Helsinki and approved by the medical ethics committee of Nanfang Hospital, Southern Medical University, China. Written informed consent was obtained from all patients before the operation. HCC tissues and the adjacent non-tumor tissues collected from 125 HCC patients (109 men and 16 women) between 2017 and 2018 were obtained from Nanfang Hospital, Southern Medical University, Guangdong Province, China. All HCC cases were confirmed by a senior pathologist and staged based on the 2011 Union for International Cancer Control TNM classification of malignant tumors.

### Cell culture

The human HCC cell lines, include MHCC97H, Hep3B, HCCLM3, Huh-7, HepG2, Bel-7404, and QGY-7701, LO2 (Normal human liver cell) were obtained from our laboratory. All the cell lines were cultured in DMEM (Gibco, USA) with 10% fetal bovine serum (FBS; Gibco, USA) in a humidified atmosphere containing 5% CO_2_ at 37 °C.

### RNA isolation and quantitative real time polymerase chain reaction (qRT-PCR)

Total RNA of cultured cells was extracted using the Ambion Trizol reagent (Thermo Fisher Scientific, Halethorpe, MD) according to the manufactures’ instructions. cDNAs were synthesized using Prime Script TM RT reagent Kit (#RR037A, TaKaRa, Dalian, China) from 500 ng of total RNA. qRT-PCR analysis of mRNA expression was performed as described previously with normalization to α-actin. The gene primers used are listed as following:

Tusc3: forward, 5ʹ-GAGAGCTGATACTTTTGACCTCC-3ʹ.

Reverse, 3ʹ-CCCGAATATGAACATCCGTTCTG-5ʹ.

LIPC: forward, 5ʹ-CCCAGTCCCCCTTCAAAGTT-3ʹ.

Reverse, 3ʹ-CAGCTCGCCGATATCCACAT-5ʹ.

Actin: forward, 5ʹ-CTCCCTGGAGAAGAGCTACGAGC-3ʹ.

Reverse, 3ʹ-CCAGGAAGGAAGGCTGGAAGAG-5ʹ.

### Protein extraction and western blotting

The protein lysate was obtained by scraping the cells in cold PBS. After centrifugation, PBS was abandoned, and the cells were incubated in lysis buffer (Fdbio Science, China) for 30 min on ice. Then the mixture was centrifuged for another 30 min, and the supernatant was collected as protein lysate. Protein levels were examined by western blotting using the antibodies as follows (Table 1). And protein expression was detected by chemiluminescence (ECL, Pierce). Expression of β-tubulin (Proteintech) was used as a protein loading control.

**Table 1:** The reagents used in the study

Antibody	Source	Identifiers
TUSC3	Proteintech, China	1:800, #16039
LIPC	Proteintech, China	1:500, #21133
β-tubulin	Proteintech, China	1:2000, #10068
pAKT	Cell Signaling Technology, America	1:1000, #9271
AKT	Cell Signaling Technology, America	1:1000, #9272
E-cadherin	Proteintech, China	1:1000, #20874
N-cadherin	Proteintech, China	1:1000, #22018
Vimentin	Proteintech, China	1:1000, #10366
MK2206 inhibitor	Proteintech, China	5 mg, #S1078

### Immunohistochemistry assay

The immunohistochemistry (IHC) analysis was performed using the streptavidin-perosidase (SP) method. The sections were deparaffinised and rehydrated, and endogenous peroxidase was inhibited with 0.3% H_2_O_2_ methanol (AMRESO) for 15 min. For antigen retrieval, slides were boiled in sodium citrate buffer (0.01 M, pH 6.0) for 5 min in a pressure cooker. After blocking with the 10% normal goat serum, the primary antibodies (rabbit anti-TUSC3, 1:100 dilutions; rabbit anti-LIPC, 1:100 dilutions; Proteintech, China) in blocking buffer were applied and the slides were incubated at 4 °C overnight. After 3× PBS washes, the sections were treated with horseradish peroxidase (HRP)-conjugated anti-mouse/rabbit IgG (1:2000, #7074, Cell Signalling, Danvers, MA). Finally, the visualization signal was developed with 3-3′-diaminobenzidine-hydrogen peroxide (Maixin, Fuzhou, China), and the slides were counterstained in haematoxylin.

The total TUSC3 immunostaining score was calculated as the sum of the percentage positivity of stained tumor cells and the staining intensity. The positive percentage was scored from 0 to 4, with 0 for < 0%, 1 for 1–25%, 2 for 26–50%, 3 for > 51–75% and 4 for > 75%. The staining intensity was scored from 0 to 3, with 0 for no staining, 1 for weakly stained, 2 for moderately stained, and 3 for strongly stained. Then the whole score of TUSC3 expression was calculated with the value of the positive percentage score multiplied by staining intensity score, ranging from 0 to 12. The final expression level of TUSC3 was defined as “negative” (0–3), “low” (4–6), “median” (7–9) and “high” (10–12). As with the TUSC3 immunostaining score, the LIPC immunostaining score was calculated in the same way.

### Transfection assays

Overexpression and down-regulation of TUSC3 were performed by lentiviral delivery using the pEZ-Lv105 vector and the psi-LVRH1GP shRNA lentiviral vector (GeneCopoeia, CA) containing TUSC3 shRNA and HEK293T packaging cell line. TUSC3 silenced MHCC97H and Hep3B cell lines, and TUSC3 overexpressed Bel-7404 and QGY-7701 cell lines were constructed. Cells transduced with empty lentiviral vectors were used as negative controls of TUSC3-overexpressed cells. Cells transduced with scrambled shRNA were used as negative controls of TUSC3-silenced cells. Recombinant lentiviruses were produced by transient transfection in 293 T cells using the calcium phosphate method. The transfectants were selected using puromycin reagent (5 μg/ml) for 2 weeks. And the transfective efficiencies were detected through western blotting and qRT-PCR analyses using the protein and mRNA samples.

For the generation of LIPC-overexpressed cells, we purchased the human LIPC expression plasmid from *GeneCopoeia* (Guangzhou, China). Bel-7404 and QGY-7701 cell lines were transfected with pcDNA3.1-LIPC using Lipofectamine 2000, and transfected cells were selected in the cell culture medium containing 2 μg/ml puromycin. Also, we performed LIPC knockdown in MHCC97H and Hep3B cell lines. The siLIPC was performed with 50 nM of siRNA using Lipofectamine RNAiMAX (Thermmo Fisher Scientific) according to the manufacturer’s instructions. siRNAs were obtained from Ruibo Biotechnology Co. (Guangzhou, China). The siRNA sequences used in the study are: siLIPC-1, si-LIPC-2, siLIPC-3, siControl. All the transfective efficiencies were detected through western blotting and qRT-PCR analyses using the protein and mRNA samples.

### Colony formation assay

Cells were seeded in 6-well plates at a density of 1 × 10^3^ per well. After 2 weeks, the cells were fixed in formalin and stained with crystal violet. The number of colonies containing ≥ 50 cells was counted under a microscope. The experiment was performed with three replicates for each cell line.

### Cell counting kit-8 assay (CCK8)

1 × 10^3^ cells/well of HCC cell lines or the control cells were seeded in 96-well plate. At 0, 1, 2, 3, 4, 5 days, 10 μl CCK8 solution (Dojindo, Tokyo, Japan) with 90 μl culture medium was added into each well and mixed for 2 h. The Microplate Autoreader (BioTek, Winooski, VT, USA) was used to measure optical density at 450 nm.

### Cell wound healing assay

Cells were seeded on six-well culture plates and incubated for 24 h (80–90% confluence). After two washes with PBS, scratch wounds were produced in each well using a 10 μl pipette tip. Migration was monitored for up to 48 h and wound margins were photographed. Images were captured using an image-analyzing frame-grabber card (LG-3 Scientific Frame Grabber; Scion, Frederick, MD, USA) and analyzed with image analysis software (Image J). Cell motility was quantified by measuring the distance between the advancing margins of cells in three randomly selected microscopic fields (× 200) at each time point.

### Transwell assay

About 1 × 10^5^ cells mixed in 200 μl serum-free media were placed in the upper compartment of 8-μm-pore transwells (Costar, Corning, Cambridge, MA, USA) and 400 μl of 10% FBS in free medium (Gibco, Invitrogen, Carlsbad, CA, USA) was added to the lower compartment. And we have checked the migration every 12 h. The cells were allowed to migrate within 72 h. For quantification, the cells in the lower compartment were stained with crystal violet and counted in five randomly chosen fields (× 200) under a light microscope. The experiment was conducted with three replicates.

### In vivo tumor growth assay

Nude mice (aged 3–4 weeks) were purchased from the Experimental Animal Center of the Southern Medical University, Guangzhou, and housed in a pathogen-free facility. To figure out the impact of the aberrant TUSC3 level on the tumor genesis, 1 × 10^6^ cells from the transfected HCC cell lines together with their control groups were injected subcutaneously into the flanks of nude mice. The length and width of tumors were measured every week with a caliper to calculate the tumor volume using the formula: V = L × W2/6(V, volume; L, length; W, width)). At the endpoint, the xenograft tumors were isolated and processed for HE assays. All experimental procedures were performed in accordance with protocols admitted by the Institutional Animal Care and Research Advisory Committee of Southern Medical University.

### Immunofluorescence assays

Cells were seeded evenly on the confocal dish at a density of 5 × 10^4^ per well for 48 h and then probed with primary antibodies against TUSC3 (#SAB4503183, Proteintech, China). Next, the coverslips were incubated with fluorescein isothiocyanate (FITC)-conjugated goat antibodies against rabbit IgG (anti-rabbit IgG, #ab6940, Abcam, MA). From then on, the dishes were protected from light. After washing with PBS in a dark place, the dishes were incubated with primary antibody against LIPC (#3538, Proteintech, China), and then incubated with rhodamine-conjugated goat antibodies against rabbit IgG (anti-rabbit IgG, #ab6940, Abcam, MA). Following counterstaining with 4′,6-diamidino-2-phenylindole (DAPI, Sigma, MO), images were captured using an Olympus FV1000 confocal laser-scanning microscope (Olympus America Inc., NY).

### Co-immunoprecipitation

For co-immunoprecipitation, cell lysates were prepared as described above from the HCC cell line MHCC97H. The cell lysates were pre-cleared by incubating with pre-blocked Protein A Sepharose beads (Zymed, San Francisco, CA, USA). Then individual antibodies (TUSC3, 1:250, #SAB4503183, Proteintech, China; LIPC, 1:500, #C2206, Proteintech, China; normal rabbit IgG, (AB_2771930, Abclonal, China) were added and incubated overnight in the shaking bed at 4 °C before harvesting of complexes with protein A Sepharose (GE Healthcare, Piscataway, NJ, USA) and brief centrifugation. Binding proteins were separated with SDS/PAGE, followed by visualization using western blotting.

### Statistical analysis

All statistical analyses were performed using the SPSS 20.0 software (SPSS Inc., Chicago, IL, USA) and the data were expressed as the mean ± s.d. P < 0.05 was considered statistically significant. The relative quantification of gene expression detected by qRT-PCR was log 2 transformed and analyzed by Student’s t-test. Linear or rank correlation analysis was performed to determine the correlation between the gene expression levels. Pearson’s Χ^2^-test was used to analyze the associations of TUSC3 or LIPC with clinical pathologic features. For cell line experiments and animal assays, data was subjected to a two-tailed Student t-test or one-way ANOVA (T-test for two-group comparisons, otherwise one-way ANOVA).

## Result

### Down-regulation of TUSC3 was related to the bigger tumor size worse differentiation and an advanced BCLC stage

**Fig. 1:**
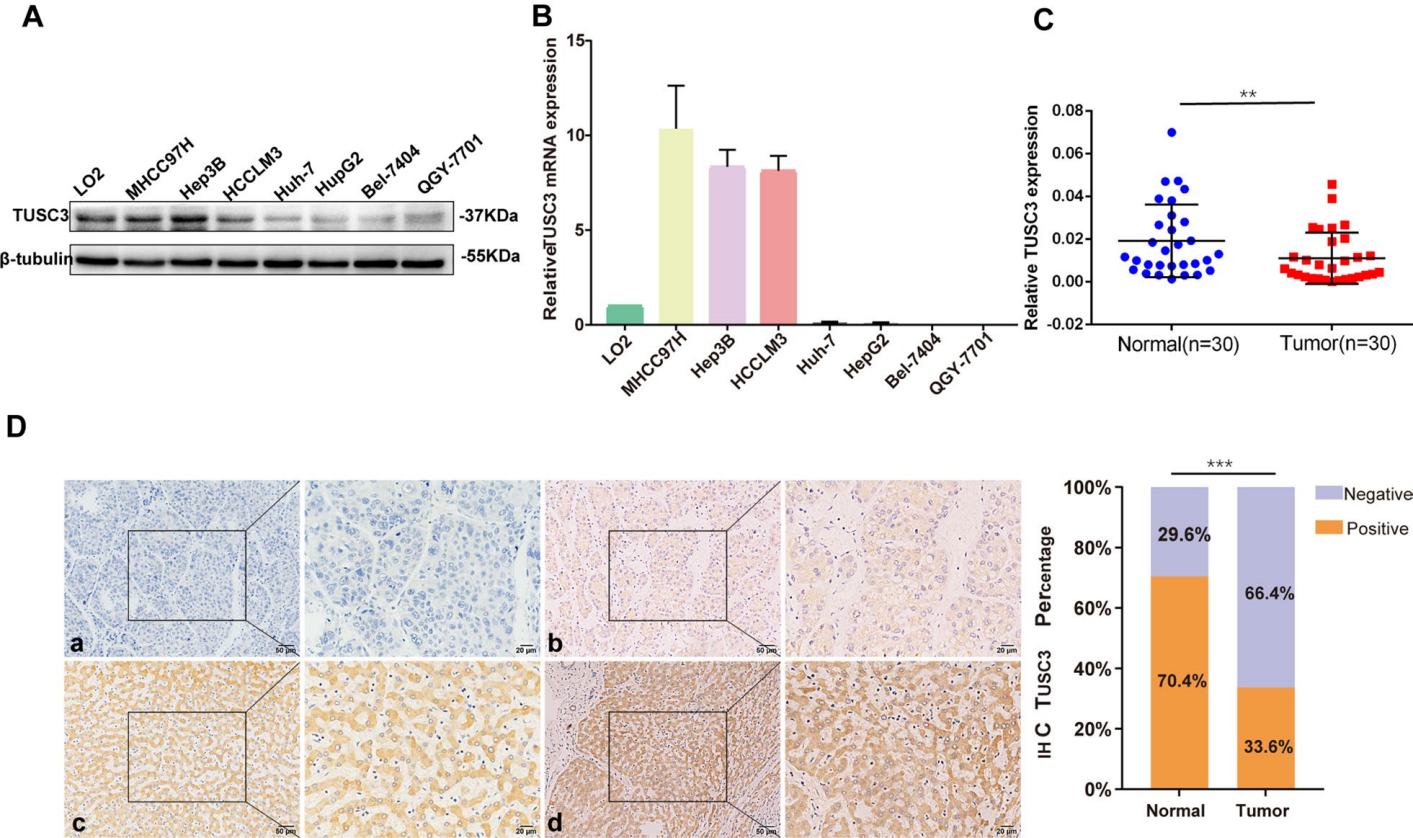
The expression of TUSC3 in HCC. The protein (**A**) and mRNA (**B**) levels of TUSC3 in one normal liver cell line (LO2) and seven HCC cell lines were detected by western blot and qRT-PCR. **C** The statistical analysis of TUSC3 mRNA levels in 30 pairs of HCC tissues and matched adjacent noncancerous tissues (**P < 0.01). **D** (Lift) Immunostaining of TUSC3 in HCC tissue samples and paired normal liver tissues. The expression of TUSC3 was defined as “negative” (a), “low” (b), “median” (c), and “high” (d) (Right) Frequency of negative, positive TUSC3 expression in HCC and adjacent normal tissue (***P < 0.001)

The expression of TUSC3 was detected by western blot (Fig. 1A) and qRT-PCR (Fig. 1B) in 7 HCC cell lines (MHCC97H, Hep3B, HCCLM3, Huh-7, HepG2, Bel-7404, QGY-7701) and a normal cell line LO2. Among the 7 HCC cell lines, TUSC3 was up-regulated at both protein and mRNA levels in MHCC97H and Hep3B cell lines, while it was down-regulated in Bel-7404 and QGY-7701 cell lines. Subsequently, we examined TUSC3 mRNA expression in 30 cases of HCC tissues and matched adjacent normal tissues. Compared to the noncancerous tissues, the expression of TUSC3 mRNA was remarkably lower in HCC tissues than in normal tissues (P = 0.0055, Fig. 1C). The immunohistochemistry analysis revealed TUSC3 protein expression in 125 surgical specimens taken from HCC patients. The results showed that TUSC3 protein was mainly localized in the cellular cytoplasm. In 125 HCC tissue samples, TUSC3 showed positive expression in 42 cases (33.6%) and low expression in 83 cases (66.4%). In 125 paired normal tissues, TUSC3 showed high expression in 88 cases (70.4%) and low expression in 37 cases (29.6%) (Fig. 1D). In addition, the expression level of TUSC3 protein was significantly lower in HCC tissues than in normal tissues (P < 0.001, Chi Square). To investigate the correlation of aberrant TUSC3 expression with HCC prognosis, the expression level of TUSC3 was statistically analyzed with the clinical pathological characteristics of HCC patients. TUSC3 expression was significantly decreased in primary tumors from HCC patients with bigger tumor size (P = 0.001, Table 2), worse differentiation (P = 0.006, Table 2) and an advanced BCLC stage (P = 0.026, Table 2), while there were no significant correlations between TUSC3 expression and age, gender, tumor number, vascular invasion, tumor capsule, AFP, HBsAg and Child–Pugh grade (Table 2). The above results indicated that the decreased expression of TUSC3 was associated with the malignant process of HCC.

**Table 2:** Relationship between TUSC3 expression and clinicopathological features of 125 patients with HCC

TUSC3 expression					
Features	n	Negative	Positive	χ^2^	*P* value
Age (years)					
≤ 50	49	36 (73.5%)	13 (26.5%)	1.805	0.179
> 50	76	47 (61.8%)	29 (38.2%)		
Gender					
Male	109	71 (65.1%)	38 (34.9%)	0.608	0.435
Female	16	12 (75.0%)	4 (25.0%)		
Tumor size (diameters) (cm)					
≤ 5	64	34 (53.1%)	30 (46.9%)	10.359	0.001
> 5	61	49 (80.3%)	12 (19.7%)		
Number of tumors					
= 1	93	59 (63.4%)	34 (36.6%)	1.426	0.232
> 1	32	24 (75.0%)	8 (25.0%)		
Differentiation					
Poor	35	28 (80.0%)	7 (20.0%)		
Moderate	75	50 (66.7%)	25 (33.3%)	10.255	0.006
High	15	5 (33.3%)	10 (66.7%)		
Vascular invasion					
Absent	60	39 (65.0%)	21 (35.0%)	1.101	0.750
Present	65	44 (67.7%)	21 (32.3%)		
Tumor capsule					
Missing	20	13 (65.0%)	7 (35.0%)	0.021	0.885
Complete	105	70 (66.7%)	35(33.3%)		
AFP (ug/l)					
≤ 20	51	31 (60.8%)	20 (39.2%)	1.218	0.270
> 20	74	52(70.3%)	22 (29.7%)		
HBsAg					
Negative	22	15 (68.2%)	7 (31.8%)	0.038	0.845
Positive	103	68 (66.0%)	35 (34.0%)		
Child–Pugh grade					
A	101	64 (63.4%)	37 (36.6%)	2.170	0.141
B	24	19 (79.2%)	5 (20.8%)		
BCLC stage					
A–B	30	15 (50.0%)	15 (50.0%)	4.759	0.029
C–D	95	68 (66.4%)	27(33.6%)		

### Knockdown of TUSC3 promoted the proliferation and migration abilities of HCC cells in vivo and in vitro

**Fig. 2:**
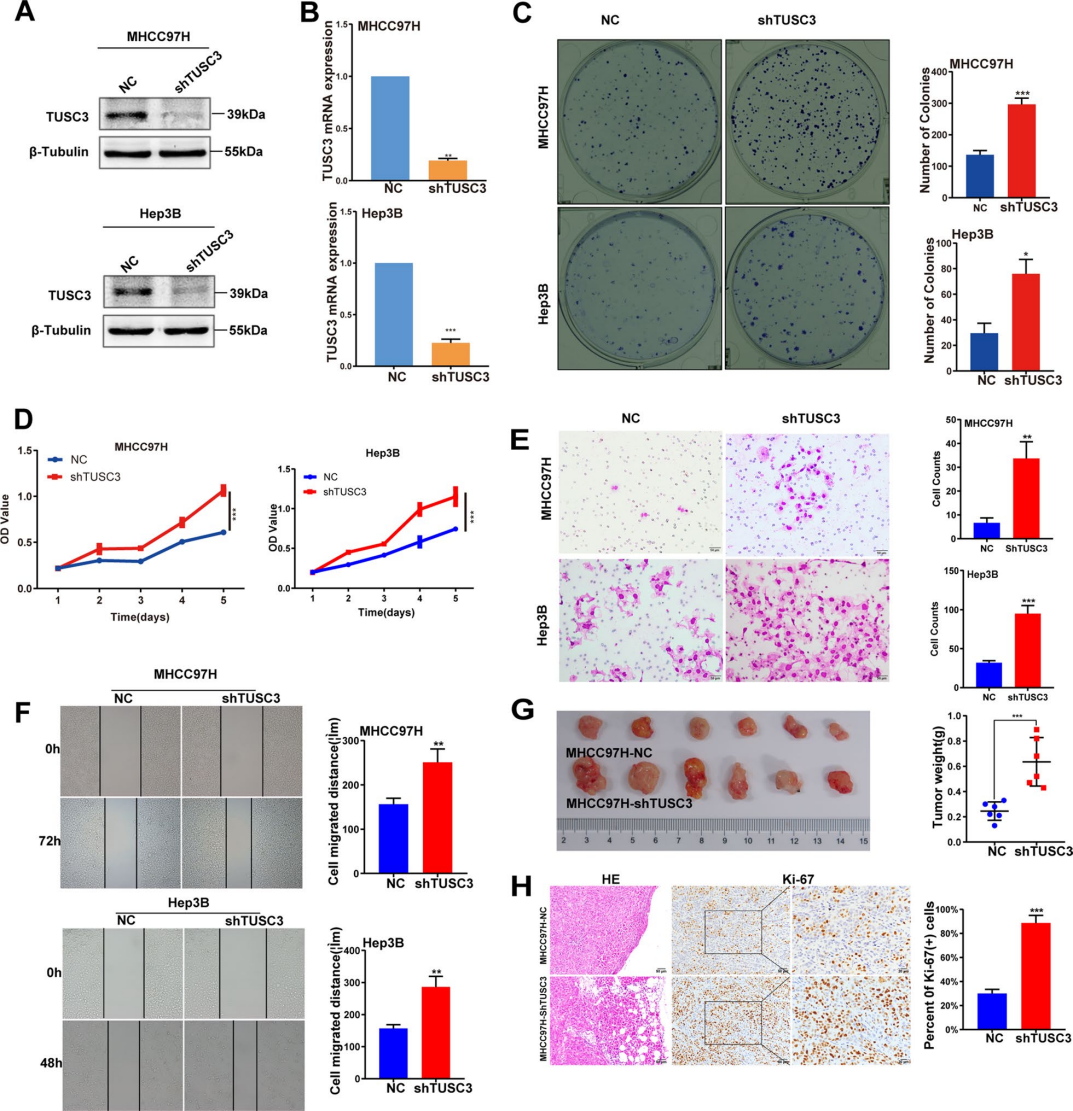
Effects of TUSC3 knockdown on the biological behavior of hepatocellular carcinoma cells. Western blot (**A**) and qRT-PCR (**B**) analysis confirmed ectopic expression of TUSC3 in stable TUSC3-transfected MHCC97H and Hep3B cells. Effect of TUSC3-knockdown both on HCC cells proliferation and clonogenicity were evaluated by plate colony formation assay (**C**) and CCK8 (**D**). The effect of TUSC3-knockdown on the migration of cells were detected by transwell migration (**E**) and wound-healing assay (**F**). **G** Representative images of subcutaneous tumor volumes in rude mice injected with the indicated cells. **H** HE and Ki-67 staining of subcutaneous tumor tissue in nude mice after being injected with MHCC97H-NC and MHCC97H-shTUSC3 cells

To reveal the role of TUSC3 in HCC, the expression of TUSC3 was knocked down in two cell lines, MHCC97H and Hep3B. The knockdown efficiency was confirmed by western blot analysis (Fig. 2A) and qRT-PCR (Fig. 2B). The results of CCK8 and colony-formation assays indicated that the reduction of endogenous TUSC3 increased the proliferative ability of MHCC97H and Hep3B cells (Fig. 2C, D). Subsequently, transwell assays and wound healing assays were performed to explore the function of TUSC3 in cell migration. The results showed that downregulation of TUSC3 promoted cell migration (Fig. 2E, F), compared to the control cells. Furthermore, we investigated the role of TUSC3 in tumorigenesis in vivo. Stable transfected MHCC97H-shTUSC3 and MHCC97h-NC cells were respectively implanted subcutaneously into nude mice, and the growth of resultant primary tumors was observed and recorded in the following days. At day 22, the tumor size in the MHCC97H-shTUSC3 group was larger than the control group, with an average size of 839.442(± 329.756) mm^3^ compared with 308.856(± 159.291) mm^3^ (P < 0.05; Fig. 2G). HE staining was applied to evaluate the histological morphology of the xenograft tumors. There was no significant difference in microscope description among the two groups, but the infiltration of tumor margin into surrounding tissue was more obvious in the MHCC97H-shTUSC3 group (Fig. 2H). Ki-67 result showed a higher proliferation index in the MHCC97H-shTUSC3 group (Fig. 2H). In summary, the results indicated that knockdown of TUSC3 promoted the proliferation and migration abilities of HCC cells in vivo and in vitro.

### Over-expression of TUSC3 suppressed the proliferation and migration abilities of HCC cells in vivo and in vitro

**Fig. 3:**
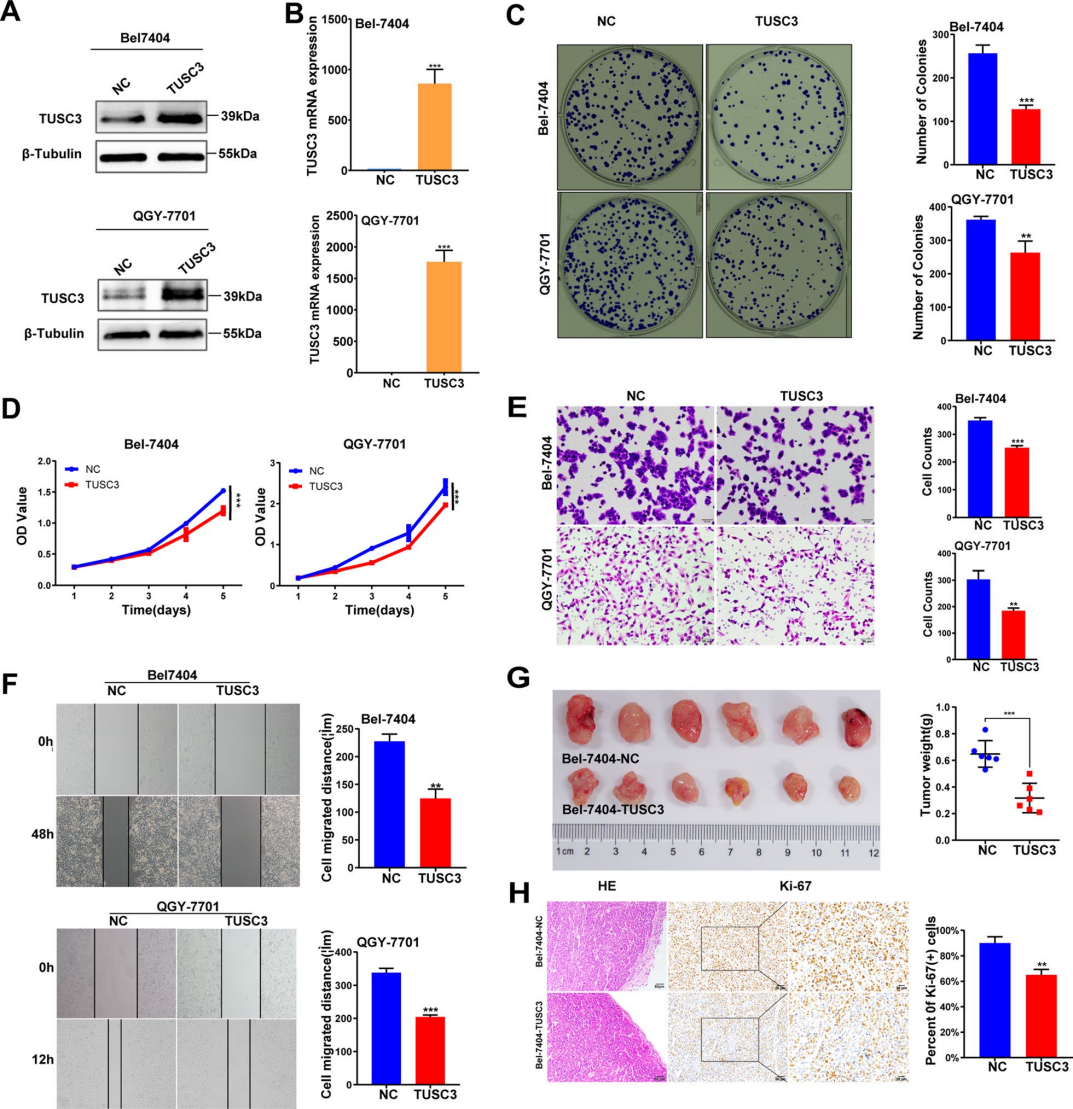
Effects of TUSC3 overexpression on the biological behavior of hepatocellular carcinoma cells. Western blot (**A**) and qRT-PCR (**B**) analysis confirmed ectopic expression of TUSC3 in stable TUSC3-transfected Bel-7404 and QGY-7701 cells. The effect of TUSC3-knockdown both on HCC cells proliferation and clonogenicity were evaluated by CCK8 (**C**) and plate colony formation assay (**D**). Effect of TUSC3-knockdown on migration of cells were detected by transwell migration assay (**E**) and wound-healing assays (**F**). **G** Representative images of subcutaneous tumor volumes in rude mice injected with indicated cells. **H** HE and Ki-67 staining of subcutaneous tumor tissue in nude mice after injected with Bel-7404-NC and Bel-7404-TUSC3 cells

To further evaluate the biological effects of TUSC3, we performed gain-of-function studies in the HCC cells, Bel-7404 and QGY-7701. The overexpression efficiencies were confirmed by western blot assays (Fig. 3A) and qRT-PCR (Fig. 3B). Overexpression of TUSC3 led to the worse cell growth and migration ability in HCC cell lines. Both Bel-7404-TUSC3 and QGY-7701-TUSC3 HCC cells decreased the capacity of proliferation compared with the control group in colony formation and CCK8 assays (Fig. 3C, D). Moreover, lower migration rates and fewer wound closures were observed in Bel-7404-TUSC3 and QGY-7701-TUSC3 HCC cells compared with the control group, revealed by the transwell migration assay and wound healing assay (Fig. 3E, F). Subsequently, stable Bel-7404-TUSC3 or Bel-7404-NC cells were injected into the groins of the nude mice subcutaneously, and all the diameters of the tumor were recorded during the observation time. The results showed that tumors taken from the Bel-7404-TUSC3 group were smaller than those from the Bel-7404-NC group (Fig. 3G). Besides, Ki-67 staining showed that the tumor cells of the Bel-7404-NC group showed a higher proliferation index in comparison with the Bel-7404-TUSC3 group (Fig. 3H). All the above results indicated that the overexpression of TUSC3 inhibited HCC cells proliferation and migration in vivo and in vitro. So, TUSC3 could act as a suppressor in HCC development.

### LIPC was down-regulated and positively associated with TUSC3 expression in HCC

**Fig. 4:**
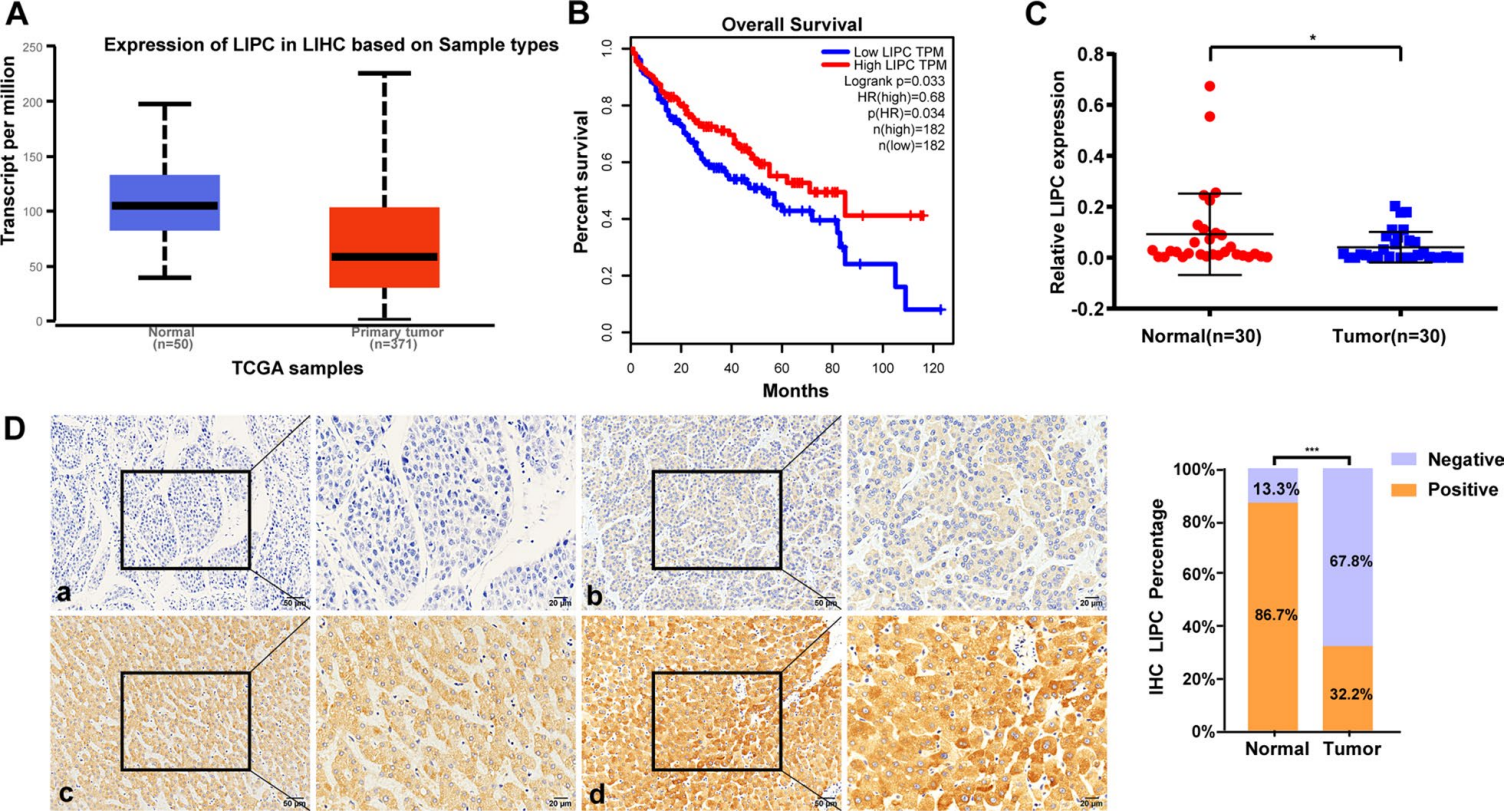
Expression of LIPC in HCC tissues. **A** Analysis of LIPC expression differences in hepatocellular carcinoma and normal liver tissue by UALCAN online database. **B** Kaplan–Meier overall survival curve of two HCC groups: LIPC low expression and high expression. **C** The statistical analysis of LIPC mRNA levels in 30 pairs of HCC tissues and matched adjacent noncancerous tissues. (*P < 0.05). **D** (Lift) Immunostaining of LIPC in HCC tissue samples and paired normal liver tissues. The expression of LIPC was defined as “negative” (a), “low” (b), “median” (c), and “high” (d); (Right) Frequency of negative, positive LIPC expression in HCC and adjacent normal tissue (***P < 0.001)

Lipase C hepatic type (LIPC) was the most related gene to TUSC3 through the analysis of the TUSC3 microarray. In order to explore the role of LIPC in HCC, the GEPIA database was used to analyze the expression of LIPC in HCC. The results showed that the expression of LIPC was down regulated in HCC (Fig. 4A). Next, the relationship between LIPC mRNA and the survival rate of HCC patients was analyzed via the Kaplan–Meier Plotter, and the result showed that the survival rate of the low LIPC expression group was lower than that of the high LIPC expression group (Fig. 4B). After checking the expression pattern of LIPC in tumor tissues from HCC patients, the expression level of LIPC mRNA in 30 cases of HCC tissues was lower than that in matched non-cancerous tissues (Fig. 4C). Subsequently, IHC was performed on 90 cases of HCC tissues and paired normal tissues and showed that LIPC was detected in the cellular cytoplasm. In 90 HCC tissue samples, LIPC showed weakly positive expression in 29 cases (32.2%) and negative expression in 61 cases (67.8%). In 90 paired normal tissue, LIPC showed strongly positive expression in 78 cases (86.7%) and negative expression in 12 cases (13.3%) (Fig. 4D). In addition, the expression level of LIPC protein was significantly lower in HCC tissues than in normal tissues (P < 0.001, Chi Square). The expression level of LIPC was statistically analyzed with clinical pathological characteristics of HCC patients. LIPC expression was greatly decreased in primary tumors from HCC patients with bigger tumor size (P = 0.006, Table 3), more tumor amounts (P = 0.023, Table 3), worse differentiation (P = 0.003, Table 3), higher AFP level (P = 0.043, Table 3) and an advanced BCLC stage (P = 0.024, Table 3), while there were no significant correlations between LIPC expression with age, gender, vascular invasion, tumor capsule, HBsAg and Child–Pugh grade (Table 3). According to the results, the decrease of LIPC expression was linked to a worse prognosis in HCC patients.

**Table 3:** Relationship between LIPC expression and clinicopathological features of 90 patients with HCC

LIPC expression					
Features	n	Negative	Positive	χ^2^	*P* value
Age (years)					
≤ 50	34	27 (79.4%)	7 (20.6%)	3.386	0.066
> 50	56	34 (60.7%)	22 (39.3%)		
Gender					
Male	79	53 (67.1%)	26 (32.9%)	0.141	0.708
Female	11	8 (72.7%)	3 (27.3%)		
Tumor size (diameters) (cm)					
≤ 5	40	21 (52.5%)	19 (47.5%)	7.695	0.006
> 5	50	40 (80.0%)	10 (20.0%)		
Number of tumors					
= 1	67	41 (61.2%)	26 (38.8%)	5.203	0.023
> 1	23	20 (87.0%)	3 (13.0%)		
Differentiation					
Poor	26	19 (73.1%)	7 (26.9%)		
Moderate	54	40 (74.1%)	14 (25.9)	11.767	0.003
High	10	2 (20.0%)	8 (80.0%)		
Vascular invasion					
Absent	45	28 (62.2%)	17 (37.8%)	1.272	0.259
Present	45	33 (73.3%)	12 (26.7%)		
Tumor capsule					
Missing	15	12 (80.0%)	3 (20.0%)	1.231	0.267
Complete	75	49 (65.3%)	26 (34.7%)		
AFP (ug/l)					
≤ 20	42	24 (57.1%)	18 (42.9%)	4.078	0.043
> 20	48	37 (77.1%)	11 (22.9%)		
HBsAg					
Negative	17	9 (52.9%)	8 (47.1%)	2.112	0.146
Positive	73	52 (71.2%)	21 (28.8%)		
Child–Pugh grade					
A	70	46 (65.7%)	24 (34.3%)	0.614	0.433
B	20	15 (75.0%)	5 (25.0%)		
BCLC stage					
A–B	21	10 (47.6%)	11 (52.4%)	5.097	0.024
C–D	69	51 (73.9%)	18 (26.1%)		

### LIPC negatively modulated the development of HCC

**Fig. 5:**
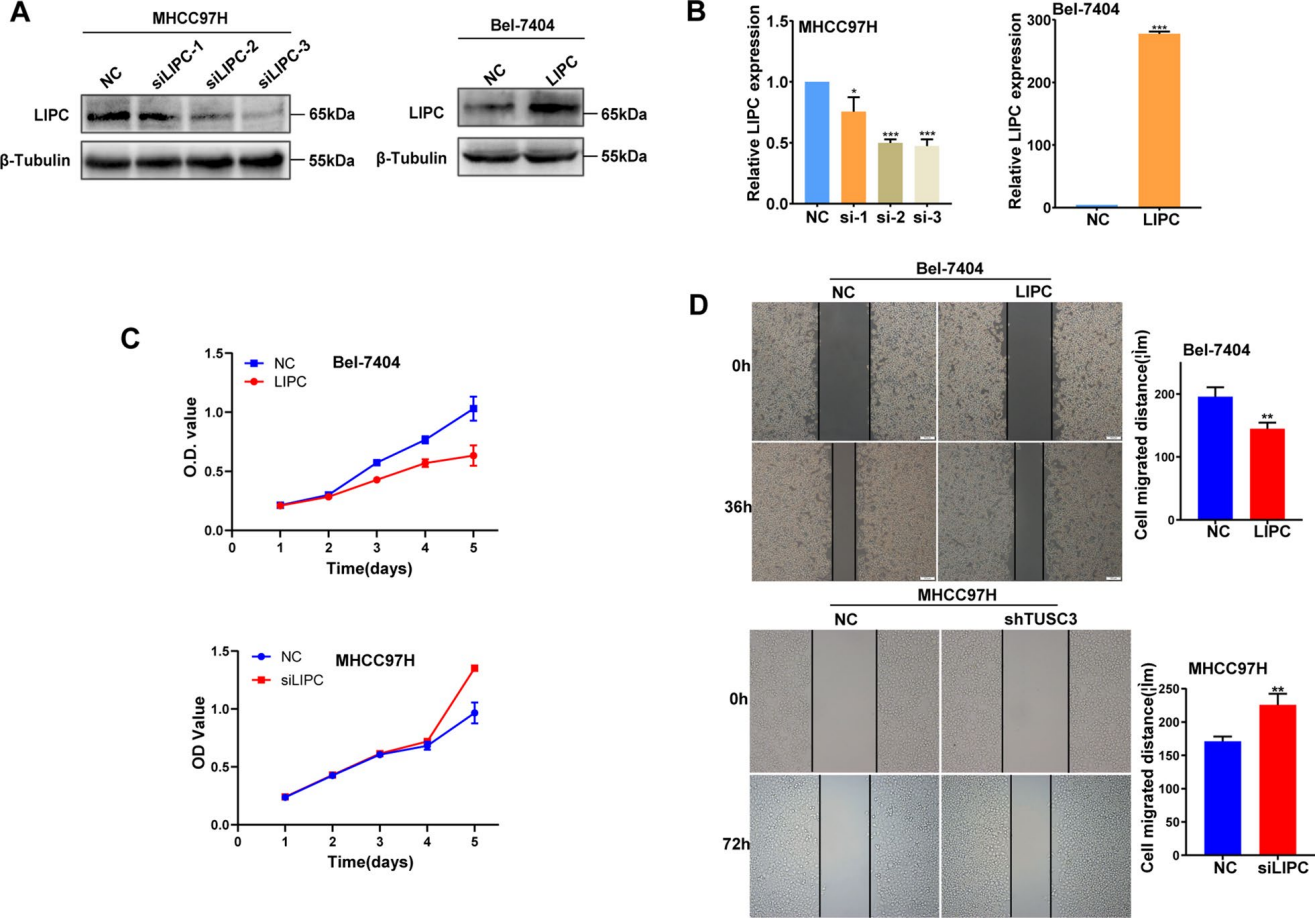
Up-regulation of LIPC inhibits the proliferation and migration of HCC cells. **A** Western blot analysis confirmed the ectopic expression of LIPC protein in siLIPC-transfected MHCC97H, and LIPC-transfected Bel-7404 cell. **B** qRT-PCR analysis confirmed the ectopic expression of LIPC mRNA both in siLIPC-transfected MHCC97H cell and LIPC-transfected Bel-7404 cell. Effect of LIPC on the proliferation and migration of HCC cells were evaluated by CCK8 (**C**) and wound-healing assays (**D**)

Gain-of-function and loss-of-function studies were performed to investigate the role of LIPC in the development of HCC. The endogenous LIPC expression was downregulated in MHCC-97H cells and the endogenous LIPC expression was upregulated in Bel-7404 cells, and the efficiency was checked by both western blotting (Fig. 5A) and qRT-PCR (Fig. 5B). CCK8 assays were used to explore the function of proliferation in HCC cell, and the results showed that down-regulation of LIPC significantly promoted HCC cell proliferation, while the opposite effect on cell proliferation was observed in LIPC-overexpressed HCC cells (Fig. 5C). Wound healing assays were applied to reveal the function of LIPC in HCC cell migration. And the down-regulation of LIPC promoted migration rates and wound closures, while lower migration rates and fewer wound closures were observed in LIPC-overexpressed HCC cells (Fig. 5D). It is inferred that LIPC acted as a negative modulator of the malignant biological behaviors of HCC.

### LIPC was associated with TUSC3

**Fig. 6:**
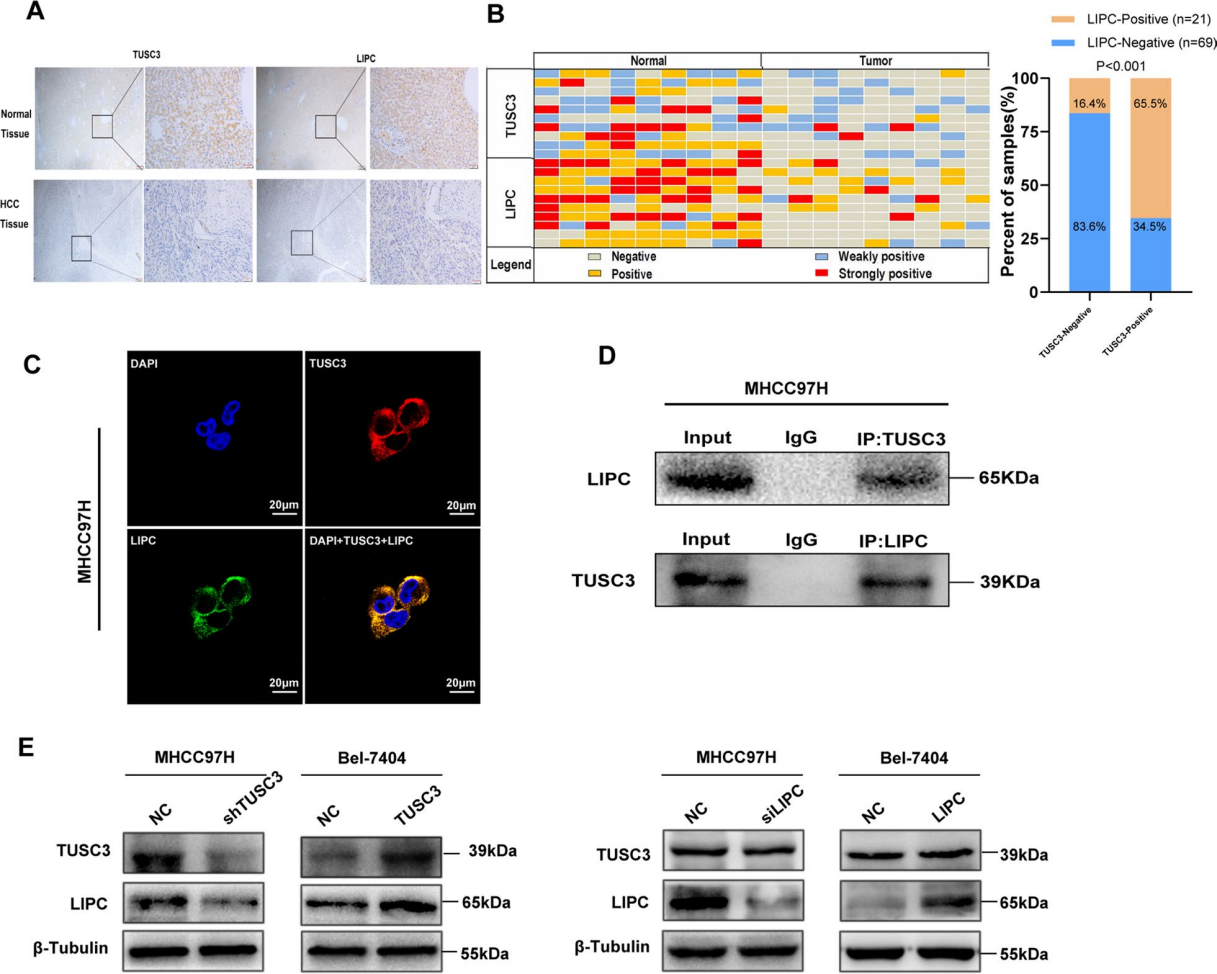
TUSC3 is associated with LIPC. **A** IHC staining of TUSC3 and LIPC in HCC tissues. **B** (Lift) Schematic diagram of Immunohistochemical scores of TUSC3 and LIPC in 90 hepatocellular carcinoma tissues and matched normal liver tissues. (Right) Pearson’s Chi-squared test was used to analyze the correlation between TUSC3 expression and LIPC in HCC patients (n = 90). χ^2^ = 50.661, P < 0.0001. **C** The expression and co-localization of TUSC3 and LIPC in HCC cell MHCC97H was detected by immunofluorescence staining. **D** TUSC3 co-immunoprecipitated with LIPC in HCC cell MHCC97H. **E** Western blot analysis the expression of LIPC in shTUSC3-transfected MHCC97H and TUSC3-transfacted Bel-7404 cell and the expression of TUSC3 in siLIPC-transfected MHCC97H and LIPC-transfected Bel-7404 cell

To figure out the relationship between TUSC3 and LIPC, the expression of TUSC3 and LIPC were evaluated and analyzed in serial sections from 90 HCC tissues (Fig. 6A). The IHC revealed that the expression of LIPC was positively correlated with TUSC3 (Fig. 6B). In addition, an immunofluorescence assay was conducted to locate the two proteins in the HCC cells, and the result showed that TUSC3 and LIPC were co-expressed and overlapped in the cytoplasm (Fig. 6C). Besides, co-immunoprecipitation analysis revealed the protein–protein interaction between TUSC3 and LIPC (Fig. 6D), which indicated that endogenous TUSC3 was physically associated with LIPC. To further explore the relationship between TUSC3 and LIPC, the expression of LIPC was detected in TUSC3-overexpressed HCC cells or TUSC3-silenced HCC cells, and the result was that the expression of LIPC was regulated by TUSC3. On the contrary, there were no impacts on TUSC3 in LIPC-overexpressed HCC cell or LIPC-silenced HCC cell (Fig. 6E). Based on the results, LIPC was down-regulated in HCC and associated with TUSC3.

### TUSC3 suppressed EMT progress through AKT signaling

**Fig. 7:**
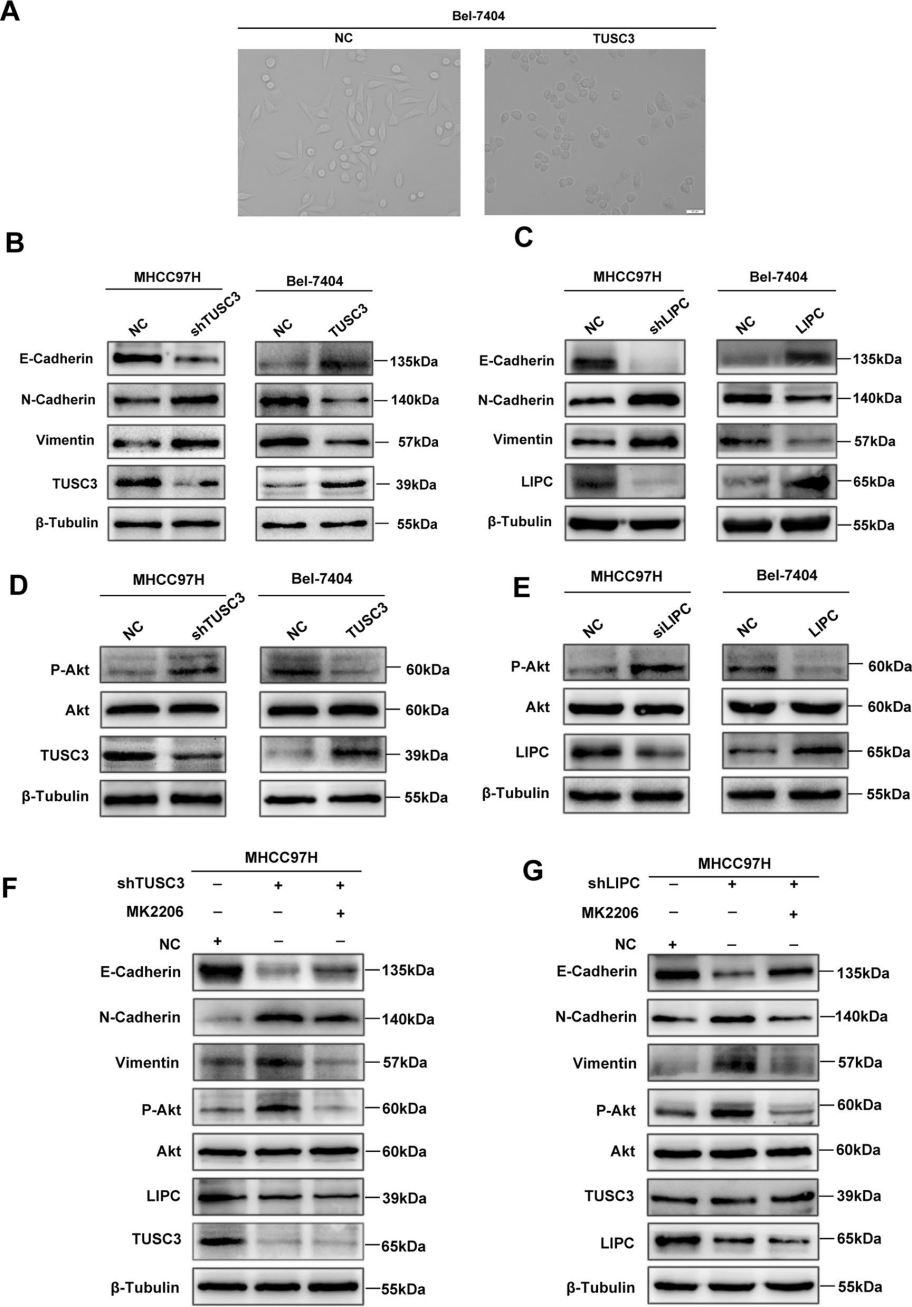
TUSC3-LIPC inhibits EMT in hepatocellular carcinoma via Akt signaling pathway. **A** Overexpression of TUSC3 alters cell phenotype; Western blot analysis the expression of EMT signaling pathway (**B**, **C**) and AKT signaling pathway (**D**, **E**) in shTUSC3-transfected MHCC97H and TUSC3-transfacted Bel-7404 cell as well as siLIPC-transfected MHCC97H and LIPC-transfected Bel-7404 cell; **F**, **G** Western blot analysis the expression of EMT signaling pathway and PI3K–AKT signaling pathway in shTUSC3-transfected or siLIPC-transfected MHCC97H cell treated with MK2206

Intriguingly, the epithelial phenotype was observed in BEL-7404-TUSC3 cells (TUSC3) and the spindle cell phenotype was observed in its control group (NC), showing epithelial-to-mesenchymal transition (EMT) inhibited by TUSC3 overexpression (Fig. 7A). To further explore the mechanism by which TUSC3 regulates the progression of HCC and to determine whether it can affect the EMT occurrence of HCC by regulating LIPC, we assessed the expression of EMT markers by western blot assays after TUSC3 or LIPC overexpression/knockdown. The results showed that the expression levels of N-cadherin and vimentin were both up-regulated and the expression levels of E-cadherin were down-regulated in TUSC3 or LIPC knockdown cell lines (Fig. 7B, C). On the contrary, TUSC3 or LIPC overexpression was identified to increase the protein level of the epithelial marker E-cadherin as well as repress the levels of the mesenchymal markers, N-cadherin and vimentin (Fig. 7B, C). Then, we further examined the AKT and pAKT levels through western blotting assay, which indicated that the deficiency of TUSC3 or LIPC promoted the phosphorylation of AKT in comparison to the cells with empty vectors, while TUSC3 or LIPC overexpression had the complete opposite effect (Fig. 7D, E). The results suggest that TUSC3 and LIPC suppress the AKT signaling.

In order to determine whether TUSC3 and LIPC modulate EMT through AKT signaling, we used the AKT inhibitor MK2206 applied to TUSC3 or LIPC knockdown cells. When treated with the AKT inhibitor MK2206, a considerable reduction in phosphorylation of AKT but no influence on the TUSC3 and LIPC levels were observed via western blotting. Besides, down-regulating pAKT made contributions to the upregulation of E-cadherin along with the decrease of N-cadherin and vimentin (Fig. 7F, G). In conclusion, we conjectured that TUSC3 inhibited the proliferation and migration of HCC cells through restraining EMT via the LIPC/AKT axis.

## Discussion

In this study, the expression of TUSC3 was downregulated in HCC tissues, and the expression of TUSC3 in HCC tissues were inversely related to tumor size, degree of differentiation, and BCLC stage. These outcomes basically agreed with Sheng’s conclusion [20]. However, we did not have enough prognosis data used for survival analysis. In addition, gain-of-function and loss-of-function assays showed that TUSC3 inhibited the proliferation and migration of HCC cells. Additionally, TUSC3 may alter the activity of the LIPC/AKT axis to encourage the progression of HCC. All the above results indicated that the decline of TUSC3 expression was associated with the malignant process of HCC and that TUSC3 indeed plays a tumor suppressive role in the progression of HCC.

TUSC3 is a subunit of the oligosaccharide transferase complex with oxidoreductase activity. And it plays a very important role in catalyzing the N-terminal glycosylation of proteins [21]. Aberrant expression of TUSC3 leads to alternations in N-glycosylation, which is closely related to tumorigenesis and malignance [22]. TUSC3 has been reported in a variety of cancers, such as prostate cancer, ovarian cancer, and pancreatic cancer, and its effects on cell proliferation, migration, and invasion have also been validated [6, 7, 23]. However, the mechanism of TUSC3 in HCC has not been previously reported.

Through the analysis of the TUSC3 microarray, LIPC was the most related gene to TUSC3. Synthesized in the endoplasmic reticulum of liver parenchymal cells, LIPC containing N-linked high mannose type was transferred to Golgi and secreted as an active form [24]. Based on previous studies, LIPC plays a disparate function in different cancers [12–16]. In our study, the protein and mRNA levels of LIPC were both downregulated in HCC, and it was important to note that there was a significant correlation between the downregulation of LIPC expression and the clinicopathological traits of HCC patients, including tumor size, tumor amounts, differentiation grade, AFP level, and BCLC stage. And vitro studies showed that the downregulation of LIPC promoted the proliferation and migration of HCC cells. It was shown that the downregulation of LIPC expression was significantly associated with unfavorable progression of HCC. Moreover, the function of LIPC in the development of HCC was first demonstrated in this study.

In order to elaborate the relationship between TUSC3 and LIPC, we performed IHC, co-immunoprecipitation, immunofluorescence co-localization, and western blotting assays, and these results showed that TUSC3 can act on LIPC and positively regulate its expression. As TUSC3 can change the process of N-linked glycosylation, we speculate that TUSC3 regulates LIPC by the function of oligosaccharyltransferase. Unfortunately, the detailed mechanisms of how TUSC3 regulates LIPC need to be further studied.

The epithelial–mesenchymal transition refers to the biological process in which cells with an epithelial phenotype are transformed into cells of mesenchymal origin under the modulation of certain cytokines [25]. It was reported that TUSC3 enhances EMT progress in colorectal cancer [8] and non-small cell lung cells [26]. On the contrary, it was also reported that TUSC3 prevents the EMT process in ovarian cancer [7]. Lipid metabolism is reportedly involved in EMT in cancer, according to recent studies. Fatty acid synthetic enzymes regulated the EMT process in breast cancer [27–29]. In this study, we found that the expression of EMT-related genes was changed after the alternation of TUSC3 and LIPC expression. The results implied that TUSC3 and LIPC may be involved in EMT. The AKT signaling pathway has been demonstrated to have a substantial influence on the EMT process [30–32]. As a tumor suppressor gene, it was reported that TUSC3 is related to the progression of glioblastoma by inhibiting the activity of the Akt signaling pathway [33, 34]. In addition, previous research has demonstrated that suppressing TUSC3-dependent AKT signaling pathway may affect the progression of melanoma cells [35], prostate cancer cells [6], and cervical squamous cell carcinoma cells [36]. Therefore, the current work looked at whether the downregulation of TUSC3 may affect the AKT signaling pathway in HCC cells. In this study, we found that the activity of AKT phosphorylation was changed after the alternation of TUSC3 and LIPC expression. Furthermore, treatment of the cell with the AKT inhibitor MK2206 inhibited the expression of p-AKT and eliminated the effect of shTUSC3 or siLIPC, leading to the reversal of the EMT process. Therefore, knockdown TUSC3 resulted in the activated of AKT signalling pathway.

Altogether, downregulation of TUSC3 promoted the EMT process and HCC progression via LIPC/AKT axis.

## Conclusion

Our findings suggested that expression of TUSC3 was down-regulated and significantly associated with poor differentiation, ascent of tumor size, and high level in BCLC stage. TUSC3 could inhibit the proliferation, migration in vivo and vitro and regulate the epithelial–mesenchymal transition and Akt signaling pathway in HCC cells. In short, our study indicates that TUSC3 inhibits EMT and the progression of HCC through LIPC/AKT axis, providing a novel biomarker for diagnosis and treatment of HCC.

## Data Availability

All data generated or analyzed during this study are included in this published article.
